# Characterization of the complete mitochondrial genome of *Rhynocoris fuscipes* (Fabricius 1787) (Hemiptera: Reduviidae)

**DOI:** 10.1080/23802359.2021.2013739

**Published:** 2021-12-28

**Authors:** Juan Wang, Chuanzhen Xue, Yi Guo, Lisheng Zhang, Yuyan Li, Jianjun Mao, Mengqing Wang

**Affiliations:** a College of Plant Protection, Shanxi Agricultural University, Taigu, China; b Institute of Plant Protection, Chinese Academy of Agricultural Sciences, Beijing, China; c Plant Protection Research Institute, Guangdong Academy of Agricultural Sciences, Guangzhou, China

**Keywords:** Mitochondrial genome, Heteroptera, Reduviidae, *Rhynocoris fuscipes*

## Abstract

*Rhynocoris fuscipes* (Fabricius 1787) is an important predator in China. In current study, the complete mitochondrial genome of *R. fuscipes* is determined. The mitogenome is 15,542 bp in size and comprises of 13 protein-coding genes, 22 transfer RNA genes, 2 ribosomal RNA genes, and a control region. Gene arrangement is identical to that of the putative ancestral arrangement of insects. All protein-coding genes initiate with ATN codons and terminate with TAA codons except for *COII*, *ND4*, and *ND5* use TA or a single T residue as the termination codons. All tRNAs have the clover-leaf structure except for the *tRNA^Ser(AGN)^* and the length of them range from 62 to 70 bp. The phylogenetic result supports the monophyly of Harpactorinae and the sister relationship between *R. fuscipes* and *Rhynocoris incertis*.

*Rhynocoris* Hahn, 1834 belongs to the family Reduviidae, a large genus with 170 known species (Zhao 2008). *Rhynocoris fuscipes* (Fabricius 1787) is a common predator in China, distributed in Southeast Asia, and was massively reared in China as a biological control agent. In this study, the complete mitochondrial genome of *R. fuscipes* was sequenced and described. Adult specimens were collected from Xiongnan, Guangdong, China (25°9′66′′ N 114°28′45′′ E) in 18th May 2021 by Mengqing Wang and identified by Mengqing Wang. Specimens were deposited in the Natural Enemy Insects Museum (Accession Number: NI2021-19) of the Institute of Plant Protection, Chinese Academy of Agricultural Sciences (IPPCAAS) (Mengqing Wang, mengqingsw@163.com, Room 307, Plant Protection Building).

The total genomic DNA was extracted from the whole body of the specimen using the QIAamp DNA Blood Mini Kit (Qiagen, Germany) and stored at −20 °C until needed. The mitogenome was sequenced in BerryGenomics company used NGS. 1 μg of genomic DNA was used to generate libraries with an average insert size of 350 bp, which were sequenced using the Illumina HiSeq S6000 (San Diego, CA, United States) with 150 bp paired-end reads on one sample per flow-cell lane. A total of 17,635,709 raw paired reads were generated. The quality of all sequences was checked using FastQC (http://www.bioinformatics.babraham.ac.uk/projects/fastqc). Clean reads were assembled and annotated using the MitoZ v2.4 pipeline (Meng et al. 2019).

The complete mitogenome of *R. fuscipes* is 15,542 bp in size (GenBank accession number: MZ440304) including 37 typical insect mitochondrial genes (13 protein-coding genes, 22 transfer RNA genes, and two ribosomal RNA genes) and one control region. Gene arrangement is identical to that of the putative ancestral arrangement of insects and most assassin bugs (Cameron 2014; Chen et al. 2018; Zhao et al. 2019; Wu et al. 2020). The nucleotide composition of the whole mitogenome shows highly A + T biased. The A + T content is 72.0% (A = 40.9%, T = 31.1%, C = 16.5%, G = 11.5%) with positive AT-skew (0.14) and negative GC-skew (−0.18). Eight PCGs (*COI*, *COIII*, *ATP6*, *ND1*, *ND4*, *ND4L*, *ND6*, and *CYTB*) initiate with ATG codons, four PCGs (*COII*, *ND2*, *ATP8*, and *ND3*) initiate with ATA codons, and one PCG (*ND5*) initiate with ATT codon. The typical termination codon TAA is assigned to ten PCGs. However, *COII*, *ND4*, and *ND5* terminate with TA or a single T residue as incomplete stop codons which is common in other Reduviidae species (Liu et al. 2019; Zhao et al. 2019; Wu et al. 2020).

There are 22 tRNA genes, ranging from 62 to 70 bp in length, and all of them can be folded into typical clover-leaf secondary structure except for *tRNA^Ser(AGN)^*, the dihydrouridine (DHU) arm of which forms a loop, as is a common phenomenon in Reduviidae species (Linghu et al. 2018; Liu et al. 2019; Wu et al. 2020). The *lrRNA* is 1,321 bp in length with an A + T content of 75.6%, and the *srRNA* is 766 bp in length with an A + T content of 71.9%. The 875 bp control region is located between *srRNA* and *tRNA^Ile^* and shows a significant AT bias (68.3%).

Phylogenetic analyses of the available mitogenomic sequences among Reduviidae with Bayesian inference (BI) and maximum-likelihood (ML) methods using the sequences of 13 PCGs and two rRNAs resulted in identical tree topology (Figure 1). The BI tree was constructed with MrBayes 3.2.6 (Ronquist et al. 2012) under the CAT + GTR model selected by ModelFinder (Kalyaanamoorthy et al. 2017), and the ML tree was constructed by IQ-TREE 2.0.6 (Bui et al. 2020) under the GTR + I + G model estimated by PartitionFinder v1.1.0 (Lanfear et al. 2012). The phylogenetic tree shows Harpactorinae is monophyletic, which is also recovered in previous molecular and morphological analyses (Weirauch et al. 2014; Wu et al. 2020). The sister relationship between *R. fuscipes* and *Rhynocoris incertis* is also highly supported. The mitogenomic data of *R. fuscipes* provides a basic data for future research investigating the unclear relationships within Reduviidae.

**Figure 1.**
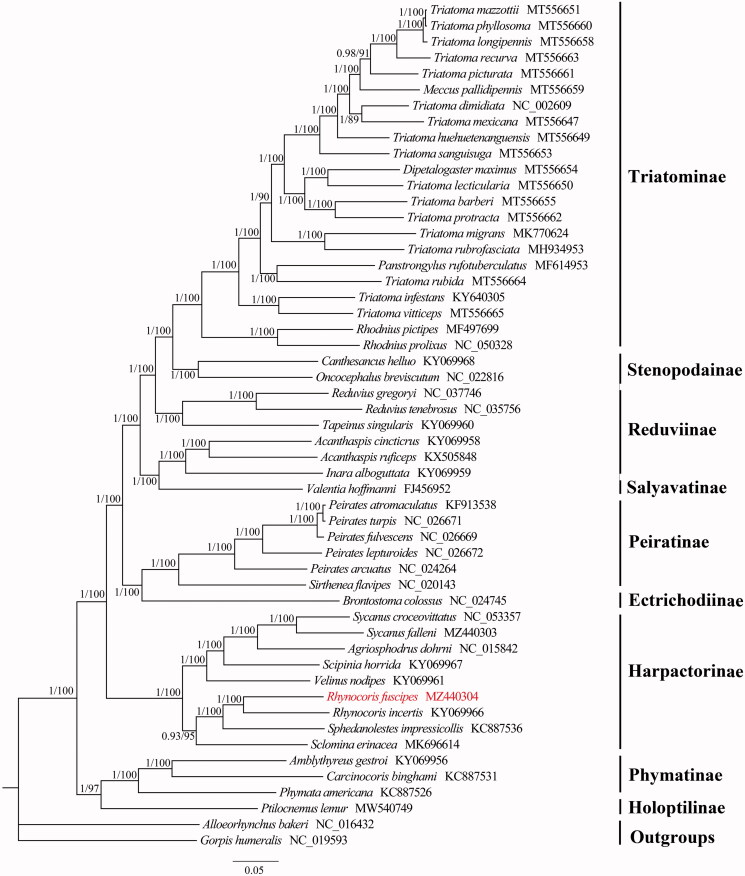
Phylogenetic relationship of 51 Reduviidae species inferred from analysis of 13 protein-coding genes and two rRNA genes. Bayesian inference and maximum-likelihood analyses recover the same three topology. Numbers on branches are Bayesian posterior probabilities (left) and bootstrap values (right). The newly sequenced mitochondrial genome was highlighted in red.

## Data Availability

The genome sequence data that support the findings of this study are openly available in GenBank of NCBI at (https://www.ncbi.nlm.nih.gov/) under the accession no. MZ440304. The associated BioProject, SRA, and Bio-Sample numbers are PRJNA740358, SRR14902174, and SAMN19842831, respectively.
